# Enhanced Hemostatic and Procoagulant Efficacy of PEG/ZnO Hydrogels: A Novel Approach in Traumatic Hemorrhage Management

**DOI:** 10.3390/gels10020088

**Published:** 2024-01-24

**Authors:** Chuyue Zhang, Yifan Wang, Yuan Xue, Junyao Cheng, Pengfei Chi, Zhaohan Wang, Bo Li, Taoxu Yan, Bing Wu, Zheng Wang

**Affiliations:** 1 Department of Orthopedics, Chinese PLA General Hospital, Beijing 100853, China; cycycyzhang301@163.com (C.Z.); wyfspine@163.com (Y.W.); xuenew301@163.com (Y.X.); cjyspine@163.com (J.C.); chipengfei301@163.com (P.C.); wzhkingkong@126.com (Z.W.); lb1995301@163.com (B.L.); yantaoxu0409@163.com (T.Y.); 2 Chinese PLA Medical School, Beijing 100853, China

**Keywords:** hemostasis, polyethylene glycol, zinc oxide, trauma care, visceral injuries

## Abstract

Managing severe bleeding, particularly in soft tissues and visceral injuries, remains a significant challenge in trauma and surgical care. Traditional hemostatic methods often fall short in wet and dynamic environments. This study addresses the critical issue of severe bleeding in soft tissues, proposing an innovative solution using a polyethylene glycol (PEG)-based hydrogel combined with zinc oxide (ZnO). The developed hydrogel forms a dual-network structure through amide bonds and metal ion chelation, resulting in enhanced mechanical properties and adhesion strength. The hydrogel, exhibiting excellent biocompatibility, is designed to release zinc ions, promoting coagulation and accelerating hemostasis. Comprehensive characterization, including gelation time, rheological properties, microstructure analysis, and swelling behavior, demonstrates the superior performance of the PEG/ZnO hydrogel compared to traditional PEG hydrogels. Mechanical tests confirm increased compression strength and adhesive properties, which are crucial for withstanding tissue dynamics. In vitro assessments reveal excellent biocompatibility and enhanced procoagulant ability attributed to ZnO. Moreover, in vivo experiments using rat liver and tail bleeding models demonstrate the remarkable hemostatic performance of the PEG/ZnO hydrogel, showcasing its potential for acute bleeding treatment in both visceral and peripheral scenarios.

## 1. Introduction

Severe bleeding in soft tissues is a highly challenging issue in both trauma emergency care and surgery, especially in cases of visceral injuries. Compared to the comprehensive clinical intervention guidelines for acute ischemic vascular diseases, interventions for the major hemorrhage are relatively simple, but if mishandled, it can be fatal in a short period of time [1]. Rapid and effective hemostasis in trauma care can significantly reduce blood loss and prevent fatal outcomes due to blood loss [2,3,4]. Traditional hemostatic methods include gauze compression, tourniquets, sponges, hemostatic powders, and surgical suturing. However, the effectiveness of these methods is significantly influenced by the location of the bleeding. For instance, while tourniquets are effective in the short term for controlling limb hemorrhage, their effectiveness is not long-lasting. Gauze compression can be somewhat effective for deep soft tissue bleeding, but its hemostatic effectiveness depends on the degree of compression and the shape of the bleeding site [5]. Hemostatic sponges and powders can evenly cover deep tissue surfaces, but their effectiveness is often diluted due to tissue movement and wet conditions, leading to recurrent bleeding [6,7,8]. Therefore, developing innovative hemostatic materials that provide rapid, effective, and biocompatible hemostasis in wet visceral tissues is crucial for effectively managing such bleeding.

Among the various materials being explored, injectable hydrogels have emerged as promising candidates due to their unique physical properties, adaptability, and compatibility with biological tissues [9]. There are numerous types of absorbable polymer-based hemostatic materials, including polyethylene glycol (PEG), polyvinyl alcohol (PVA), polylactic acid (PLA), and polycaprolactone (PCL). These materials can rapidly form gels, assisting in blood clotting and promoting wound healing, and are widely used in the research of hemostatic hydrogels [10]. PEG is highly esteemed as a classic synthetic polymer for its outstanding biocompatibility and unique physicochemical properties. The hydrogels made from PEG exhibit high hydratability, allowing them to absorb a significant amount of water and form a dense gel structure at the wound site. Notably, the mechanical properties of PEG hydrogels can be flexibly altered by adjusting the cross-linking density of the polymer network, allowing their hardness and elasticity to be tailored to different application needs [11]. Consequently, PEG hydrogels hold a broad spectrum of prospects in the field of biomedical applications. However, PEG-based hydrogels primarily control bleeding through physical adhesion and water absorption, lacking other bioactive properties. These deficiencies have motivated researchers to innovatively modify these hydrogels to achieve a more comprehensive integration of biocompatibility, pro-coagulation, hemostasis, and customizability, thus better meeting clinical demands.

PEG succinimidyl succinate (PEG-SS) is a unique derivative of PEG, characterized by its active succinimidyl succinate ester groups. These active ester groups are particularly prone to reacting with compounds containing active hydrogen, such as amines. When encountering PEG amine (PEG-NH_2_), the active ester group in PEG-SS is attacked by the amine, leading to the cleavage of the ester bond and the formation of a new covalent amide bond [12]. This process is highly specific, occurring rapidly at room temperature without needing a catalyst, demonstrating its efficiency. The use of PEG-SS in combination with PEG-NH2 has been shown to have a significant effect on wound healing and soft tissue repair [13]. Notably, the amine group in PEG-NH_2_ can also chelate with metal substrates, a feature that can further strengthen the cross-linked network within the gel, enhancing its structural integrity and endowing the hydrogel with additional functions [14,15,16].

Zinc oxide (ZnO) is renowned for its excellent biocompatibility and multifunctionality and has a long and extensive history of applications in the field of pharmaceutical development. As a vital trace element in human blood, zinc ions play a crucial role in the blood coagulation process. They activate key coagulation enzymes, such as thrombin, and promote platelet aggregation, thereby effectively facilitating the clotting process [17,18]. As a metal ion, Zn^2+^ can chelate with the amino groups present in PEG-NH_2_ [14,15,16]. This chelation not only speeds up the gelling process of hydrogels but also acts as a secondary cross-linking network, enhancing the internal structure and stability of the hydrogel. At present, ZnO has been widely used in combination with hydrogel scaffolds to promote wound healing, mainly playing its antibacterial and anti-inflammatory roles [19,20,21].

**Scheme 1:**
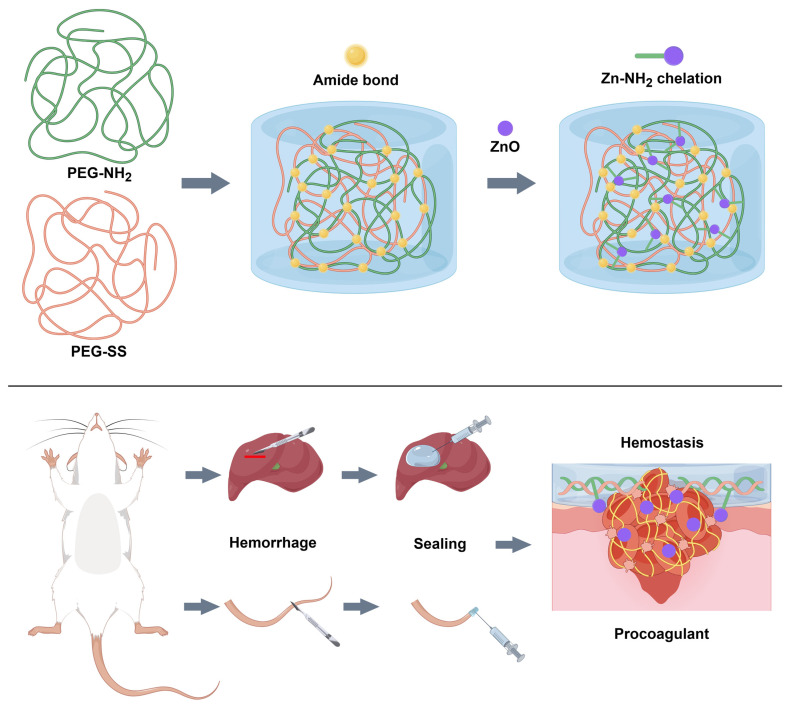
The formation mechanism of PEG and PEG/ZnO hydrogel based on amide bond and Zn-NH_2_ chelation. *By Figdraw* version 2.0.

In this study, we combined PEG-based hydrogel with ZnO to develop a dual-network structure (Scheme 1). The amide bond reaction enabled the rapid gelation to form the chemical network, while the Zn-NH_2_ chelation played a role in coordinating the physical network within the hydrogels. The coordination of the amide bond and the Zn-NH_2_ chelation contribution played a significant role in promoting the formation of a compact network, which could endow the PEG/ZnO hydrogel with a range of excellent capacities. This hydrogel, through its adhesive properties, can slowly release zinc ions at the bleeding site, effectively promoting coagulation and accelerating hemostasis. Experimental results indicate that the enhanced PEG/ZnO hydrogel exhibits superior mechanical properties and adhesion strength, especially in coagulation effects, showing significant improvement compared to traditional PEG hydrogels. After optimization, the hydrogel presented the characteristics of easy synthesis and use, excellent mechanical strength, and non-toxic composition. It functioned in hemostasis and coagulation through physical blockage and biological activity, which met both in vitro and in vivo coagulation requirements, offering a promising hemostatic solution for surgeries and emergency trauma care.

## 2. Results and Discussion

### 2.1. Preparation of Hydrogels

Different concentrations of PEG-NH_2_ and PEG-SS solution were made by dissolving different proportions (wt%) of 4-arm PEG-NH_2_ and 4-arm PEG-SS in PBS (pH 7.4). Then, PEG/ZnO and PEG hydrogels were made by mixing equal volumes of PEG solution with or without ZnO particles [12]. Take the example of the PEG3/ZnO1 hydrogel group in the following text, which includes 20 wt% PEG-NH_2_, 20 wt% PEG-SS, and 0.25 wt% ZnO.

### 2.2. Characterization of Hydrogels

**Figure 1:**
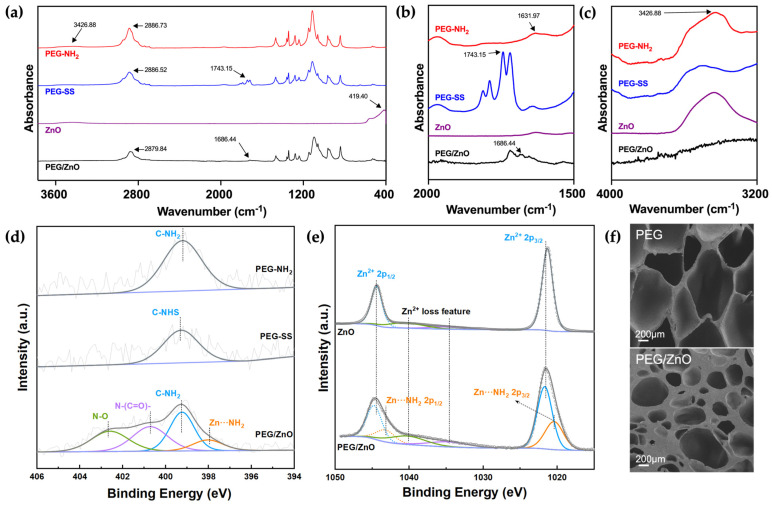
(**a**) FT-IR spectra of PEG-NH_2_, PEG-SS, ZnO, and PEG/ZnO hydrogel in the wavenumber range of 400–3600 cm^−1^. (**b**) FT-IR spectra of PEG-NH_2_, PEG-SS, and PEG hydrogel in the wavenumber range of 1500–2000 cm^−1^. (**c**) FT-IR spectra of PEG-NH_2_, PEG-SS, and PEG/ZnO hydrogel in the wavenumber range of 3200–4000 cm^−1^. (**d**) XPS spectra images of N 1 s. (**e**) XPS spectra images of Zn 2p. (**f**) SEM images of the PEG and PEG/ZnO hydrogels. Scale bar: 200 μm.

The chemical structures were analyzed using Fourier Transform Infrared Spectroscopy (FT-IR), including PEG-NH_2_, PEG-SS, ZnO, and PEG/ZnO hydrogel (Figure 1a). The hydrogel we used to test was composed of 20 wt% PEG-NH_2_, 20 wt% PEG-SS, and 0.25 wt% ZnO. As illustrated in Figure 1b,c, the absorption peak near 2800 cm^−1^ typically corresponds to C-H stretching vibrations, commonly observed in the range of 2800–3000 cm^−1^. This is primarily associated with the vibration of carbon–hydrogen bonds in methyl and methylene groups, which are prevalent in PEG compounds. For PEG-NH_2_, the N-H stretching vibration of the amino group appeared between 3300 and 3500 cm^−1^, with a peak at 3426.88 cm^−1^, while the N-H bending vibration of the amino group generally occurred in the range of 1550–1650 cm^−1^, as shown at 1631.97 cm^−1^. For PEG-SS, the absorption peak at 1743.15 cm^−1^ corresponded to the stretching vibration of C=O in the active ester group (1730–1750 cm^−1^). During the formation of PEG hydrogel, FT-IR analysis revealed the disappearance of characteristic absorption peaks at 3426.88 cm^−1^ and 1631.97 cm^−1^, indicating the involvement of amino groups (-NH_2_) in the formation of amide bonds [22]. Simultaneously, the C=O absorption peak at 1743.15 cm^−1^ in PEG-SS was replaced by a new absorption peak at 1686.44 cm^−1^ corresponding to the newly formed amide bond (C=O), which was called the amide I band [23]. These results suggested a condensation reaction between amino groups (-NH_2_) and active esters, forming amide bonds (-CONH-), which connected the PEG-NH_2_ and PEG-SS, resulting in a new covalently linked product. With the addition of ZnO into the PEG hydrogel system, Zn^2+^ underwent chelation reactions with the amino groups (-NH_2_), forming a secondary network that strengthens the hydrogel structure and accelerates gelation time. The characteristic absorption peak at 419.4 cm^−1^ for hydrogen bonding vibrations in metal oxides between zinc and oxygen ions disappeared after chelation reactions with corresponding functional groups, with the N-H absorption peak at 3426.88 cm^−1^ correspondingly weakened or disappearing due to the chelation reaction of the amino groups [24,25].

We employed X-ray Photoelectron Spectroscopy (XPS) to further investigate the characteristic elements and their existing forms in PEG-NH_2_, PEG-SS, ZnO, and PEG/ZnO hydrogels. As illustrated in Figure 1d, in PEG-NH_2_ and PEG-SS, N was present in the forms of C-NH_2_ (399.21 eV) and C-NHS (399.28 eV), respectively. In the case of PEG/ZnO, as the amine group in PEG-NH2 underwent a condensation reaction with the active ester in PEG-SS, the intensity of C-NH_2_ (400.72 eV) decreased. Concurrently, a new peak appears at 402.6 eV, indicative of the newly synthesized amide bond (-CONH-). The remaining nitrogen existed in the forms of N-O (398.03 eV) and Zn···NH_2_ (399.28 eV) [26]. Figure 1e illustrates the forms of Zn present in ZnO and PEG/ZnO. In ZnO, the spectrum of Zn 2p exhibited two peaks. The peak at 1044.4 eV corresponded to Zn^2+^ 2p_1/2_, while the peak at a lower binding energy (1021.33 eV) corresponded to Zn^2+^ 2p_3/2_. With the addition of ZnO into the PEG hydrogel, a chelation reaction occurred between Zn^2+^ and -NH_2_, resulting in two weaker peaks at 1043.22 eV and 1020.43 eV, corresponding to Zn···NH_2_ 2p_1/2_ and Zn···NH_2_ 2p_3/2_, respectively [27]. Combining the results from FT-IR and XPS, we elucidated the formation mechanism of the dual-network structure within the PEG/ZnO hydrogel.

Then, we conducted a comprehensive analysis of the impact of ZnO on the microstructure of PEG hydrogels using scanning electron microscopy. The porous structure of hydrogels is crucial for cellular migration and proliferation within a biological system [28]. As illustrated in Figure 1f, the chemical interactions among PEG molecules resulted in a uniformly porous structure in the PEG hydrogel. These interconnecting pores contributed to excellent biological properties. Upon the addition of ZnO, there was a slight reduction in pore size within the hydrogel structure [29]. The increase in cross-linking density led to a significant improvement in mechanical properties. This was correlated with the chelation reaction observed in FT-IR, where zinc ions interacted with PEG functional groups, forming a second cross-linked network, enhancing the overall structural integrity [16]. These findings suggested that the addition of ZnO can significantly influence the microstructure of PEG hydrogels, potentially altering the mechanical properties of the PEG hydrogel.

**Figure 2:**
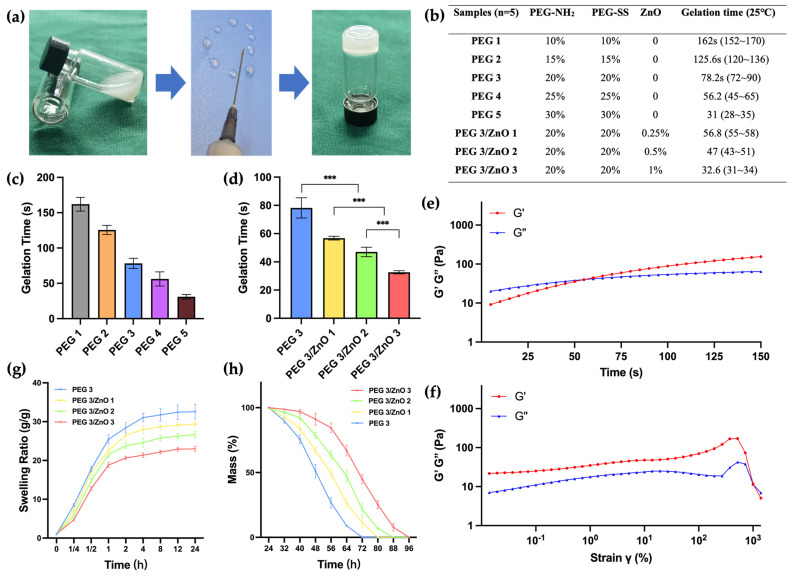
(**a**) Gelation process of the hydrogel: precursor, liquid (injectable) state, and gelation state. (**b**) Composition concentration and gelation time of PEG and PEG/ZnO hydrogels in different groups. (**c**,**d**) Gelation time of PEG and PEG/ZnO hydrogels at different concentrations (*n* = 5). (**e**) Time sweep of PEG/ZnO under an angular frequency of 6.28 rad/s and strain of 0.5%. (**f**) Strain sweep of PEG/ZnO under an angular frequency of 10 rad/s. (**g**) Swelling curves of the hydrogels (*n* = 3). (**h**) Degradation curves of the hydrogels (*n* = 3).

Achieving an appropriate gelation time is crucial for the effectiveness of hemostatic hydrogels. Prolonged gelatinization can result in delayed hemostasis, leading to extensive tissue adhesion when the liquid hydrogel precursor diffuses into neighboring healthy tissues. Conversely, a gelation time that is too short may not be conducive to practical clinical applications. To address this, we determined the gelation time of various hydrogels at 25 °C by adjusting the concentration of PEG-NH_2_, PEG-SS, and ZnO. The gelation time was assessed using the tube inversion test, as illustrated in Figure 2a. Before reaching the gelation state of the hydrogel precursor, we used a 21 G needle syringe to extract liquid composite hydrogel. All groups of hydrogels can be smoothly extruded before reaching the gelation time. The entire process was continuous and can be repeated multiple times to draw patterns [30]. The amino groups of PEG-NH_2_ can rapidly react with the succinimidyl-active ester of PEG-SS under ammonolysis conditions. Increasing the concentrations of both PEG-SS and PEG-NH_2_ from 10 wt% to 30 wt% led to a gradual decrease in gelation time from 162 s to 31 s (Figure 2b,c). The gelation time of the PEG hydrogel can be easily controlled by adjusting the concentration of PEG content. Through practical validation, we determined that a gelation time of around 50–60 s is most suitable for clinical applications, allowing for suction, injection, and in situ gelation. We found that the gelation times of PEG3 (20 wt%) and PEG4 (25 wt%) both met this optimal duration. Considering the chelation effect of zinc ions, which may accelerate the gelation process, there is a possibility that the PEG4 group exceeds the optimal gelation time. Therefore, we chose PEG3, which had a relatively lower concentration of PEG to investigate the impact of adding ZnO on the gelation time. As depicted in Figure 2d, the gelation time of PEG/ZnO hydrogels significantly decreased after the addition of ZnO. With 0.25 wt% to 1 wt% ZnO addition, the gelation time of the PEG/ZnO hydrogels reduced from 56.8 s to 32.6 s. Through chelation reactions, zinc ions can markedly enhance the gelation speed of PEG-based hydrogels, allowing for achieving the desired gelation time at lower PEG concentrations [31].

Next, we investigated the rheological properties of the hydrogel. Sweep at a fixed frequency and strain revealed the transition of the hydrogel from a liquid to a solid state. Here, G’ represents the storage modulus, reflecting the solid-like characteristics of the material, while G” represents the loss modulus, reflecting the liquid-like characteristics. As illustrated in Figure 2e, upon mixing PEG-NH_2_, PEG-SS, and ZnO, the hydrogel precursor exhibited G” > G’ for the initial 55 s, which indicated its liquid state. However, after 55 s, both G’ and G” curves reversed, which indicated the transition of the hydrogel from a liquid to a solid state. This observation aligns with the gelation time determined in our previous analysis. After gelation, we conducted a strain sweep on the hydrogel under an angular frequency of 10 rad/s. As illustrated in Figure 2f, at shear strains approached 1 × 10^3^, the molecular structure of the hydrogel experienced forces exceeding its capacity to maintain the gel state. These resulted in a transition from its original state to a liquid state, accompanied by the rupture of the hydrogel’s structure.

PEG-based hydrogels exhibit excellent swelling performance [32]. However, swelling properties have advantages and disadvantages for biological hemostatic hydrogels. The high swelling rate of hydrogels can simulate the softness and elasticity of human tissues, aiding better adaptation within the biological environment. Moreover, a high swelling rate implies superior water absorption, enabling the maintenance of a moist microenvironment in bleeding wounds and absorption of wound exudate. However, excessively high swelling rates may lead to the disruption of the hydrogel network, causing it to lose its original shape and structure. As the mechanical strength of the hydrogel decreases, both compressive strength and adhesive strength are compromised. This can be a significant issue for its use in internal hemostasis, where the decrease in hydrogel adhesion may result in recurrent bleeding. Figure 2g demonstrated that with the addition of ZnO, zinc ions chelated with amino and carboxylic anhydride groups, resulting in a more compact gel network formation in the hydrogel. This phenomenon became more pronounced with increasing ZnO concentration. Compared to PEG hydrogel, PEG/ZnO hydrogel exhibited lower swelling rates, allowing it to maintain mechanical stability during the absorption process [33]. All hydrogel groups reached a stable swelling rate after 24 h, and we measured their degradation time in vitro. The coordination structure of zinc ions provided a second network structure for PEG-based hydrogels, contributing to an extended degradation time for PEG/ZnO hydrogel. As shown in Figure 2h, the 20 wt% PEG hydrogel completely degraded after 3 days, and as the ZnO concentration increased, the degradation time gradually lengthened [12]. This indicated that a more compact network promotes more excellent stability in the hydrogel.

### 2.3. Mechanical Properties of the Hydrogels

**Figure 3:**
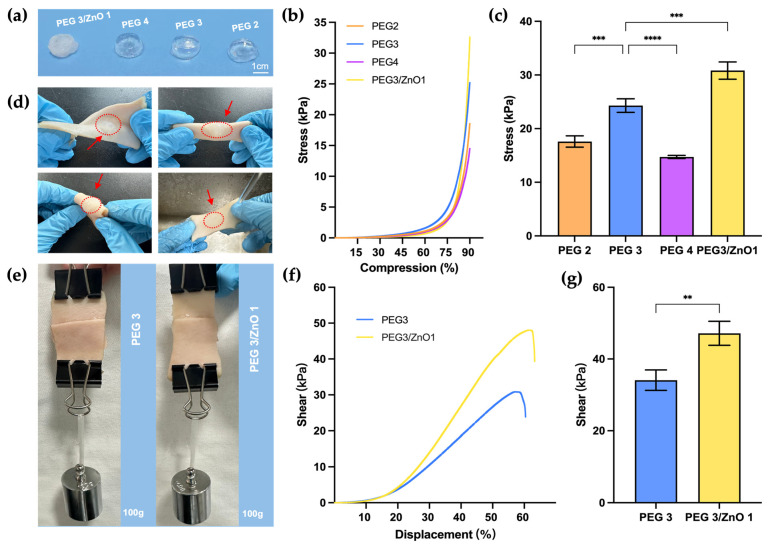
(**a**) Hydrogel samples used for compressive strength test. (**b**) Stress−compression curves of hydrogels (*n* = 3). (**c**) Compressive stress of hydrogels at 90% compression level (*n* = 3). (**d**) The porcine skin was injected with hydrogels for torsion, folding, and waterproof testing. (**e**) Adhesion of PEG and PEG/ZnO on the porcine skin. (**f**) Shear–displacement curves of different hydrogels (*n* = 3). (**g**) Shear stress of different hydrogels. (Data are presented as mean ± SD, ** *p* < 0.01, *** *p* < 0.001, **** *p* < 0.0001).

Hemostatic hydrogels need to possess robust mechanical strength to withstand pressure and prevent rupture in the body. Simultaneously, excellent adhesive strength helps maintain their position at the wound site in a moist and smooth environment inside the body, which is crucial for the reliability of hemostasis [34]. We initially selected PEG2, PEG3, PEG4, and PEG3/ZnO1 based on gelation time for compression strength testing. Figure 3a illustrates the overall appearance of the prepared hydrogel blocks, and the samples underwent compression testing on a mechanical testing machine at a rate of 2 mm/min until a deformation extent of 90%. As shown in Figure 3b,c, compared with the PEG2, PEG3, and PEG4 groups, before the concentration of PEG content reached 20 wt%, the compressive strength of the hydrogel increased with an elevation in concentration, reaching a maximum value of 24.30 ± 1.03 kPa (PEG3). Beyond this concentration, the compressive strength of hydrogels decreased with increasing PEG concentration. Since the compressive strength of PEG3 hydrogel was superior to the other two groups, we selected PEG3 with the addition of ZnO to explore the changes in mechanical properties. After adding ZnO, the compressive strength of PEG3/ZnO1 reached 30.81 ± 1.31 kPa, indicating an improvement in compressive strength. This suggests that the addition of ZnO significantly increases the cross-linking density of the hydrogel, thereby reinforcing its polymeric network structure.

Next, we selected the two hydrogel groups with the highest compression strength for further experiments. We applied PEG3 and PEG3/ZnO1 onto the surface of fresh porcine skin to assess their anti-torsion performance and waterproof properties on moist skin surfaces. As shown in Figure 3d, the hydrogel adhered tightly to the porcine skin surface under torsion and bending conditions, demonstrating resistance to the flushing action of water. Subsequently, we trimmed the fresh porcine skin into strips, applied 400 μL of PEG3 and PEG3/ZnO1 hydrogels on the respective cut ends, and then overlapped them with an overlap area of approximately 15 mm × 10 mm (Figure 3e). After allowing them to stand for 5 min, a weight of 100 g was suspended below the porcine skin. We observed that both hydrogel groups securely adhered to the fractured porcine skin and withstood the 100 g weight, preventing the porcine skin from breaking again.

To clarify the influence of ZnO on the adhesive strength of PEG-based hydrogels, we conducted further mechanical tests at a stretching speed of 10 mm/min. The test aimed to determine the maximum adhesive strength of the two hydrogels when porcine skin was stretched until rupture. As shown in Figure 3f,g, the PEG3/ZnO1 hydrogel exhibited a maximum adhesive strength of 47.16 ± 2.73 kPa, significantly higher than the maximum adhesive strength of the PEG3, which was 34.10 ± 2.34 kPa. The increase in adhesive strength may be attributed to the chelation between zinc ions and amino groups on the tissue surface. These results indicated that the addition of ZnO not only enhanced the mechanical performance of PEG-based hydrogels but also increased the adhesive strength of tissues, which was highly beneficial for adhesion in the dynamic and moist environment of internal tissues. The robust mechanical resilience exhibited by these hydrogels facilitated their stability even under dynamic tissue movements and external forces, representing a vital auxiliary factor in the attainment of hemostasis [35].

### 2.4. In Vitro Biocompatibility

**Figure 4:**
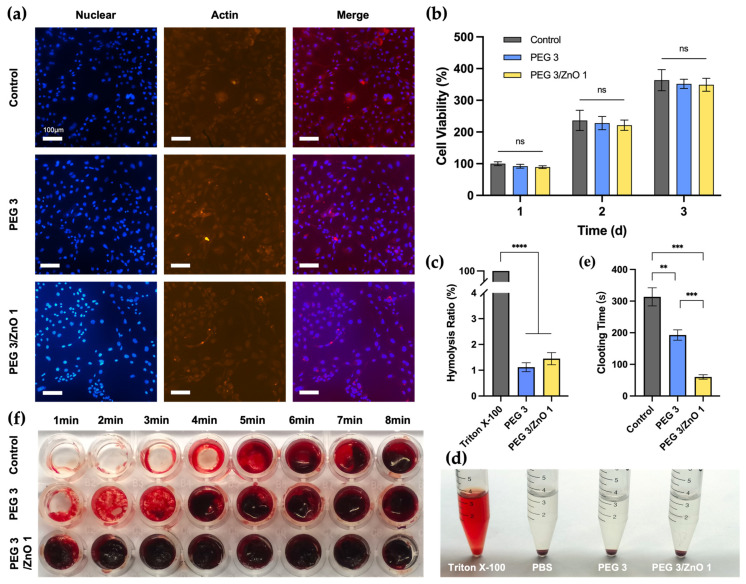
(**a**) Fluorescence staining of NIH-3T3 cells after incubation with the hydrogels for 24 h; the cell nucleus is stained blue with DAPI, and actin protein is stained red with phalloidin. Scale bar: 100 μm. (**b**) Cell viability analysis of the hydrogels for 1, 2, and 3 days. (**c**) Hemolysis ratio of the hydrogels (*n* = 5). (**d**) Hemolysis test of the hydrogels. (**e**) Clotting time of the hydrogels (*n* = 5). (**f**) Procoagulant effects of the hydrogels. (Data are presented as mean ± SD, ** *p* < 0.01, *** *p* < 0.001, **** *p* < 0.0001, ns: no significant difference).

Good biocompatibility is a prerequisite for the in vivo application of biomaterials [36]. We selected NIH-3T3 cells for the fluorescence staining and the CCK-8 assay to assess the biocompatibility of the hydrogels. Based on gelation time, compressive strength, and adhesive strength measurements, we chose PEG3 and PEG3/ZnO1 as experimental candidate groups to evaluate the impact of ZnO concentration on biocompatibility. After co-culturing cells with the hydrogel extract for 24 h, we performed fluorescence staining on NIH-3T3 cells (Figure 4a). All cells displayed a spindle-shaped morphology in all groups, with apparent nuclear staining, indicating that the hydrogel exhibits excellent biocompatibility. At the same time, the quantitative analysis results (Figure 4b) showed that cells in the PEG3 and PEG3/ZnO1 groups exhibited a significant proliferation trend within three days, with no statistically significant differences between each group. On the first day, the cell survival rates for both the PEG and PEG/ZnO groups were above 90%. Moreover, by the third day of culture, in comparison to the first day, the cell survival rates for the PEG and PEG/ZnO groups reached 352 ± 12% and 349 ± 17%, respectively. The hydrogel’s excellent adhesive properties and appropriate porosity contribute to its outstanding biocompatibility.

### 2.5. In Vitro Hemocompatibility and Procoagulant Ability

Due to the hydrogel’s need to remain in the tissue after hemostasis, the blood compatibility of this material is crucial [37]. We co-cultured rat red blood cells with 0.1% Triton X-100, PBS, PEG3 hydrogel extract, and PEG3/ZnO1 hydrogel extract for 12 h (Figure 4c,d). The appearance of the samples after centrifugation is shown in Figure 4d. Like the negative control group (PBS group), the supernatant of the PEG3 and PEG3/ZnO hydrogel groups was clear, while the supernatant of the positive control group (Triton X-100) was bright red. The hemolysis rate of each group was measured by a spectrophotometer at a wavelength of 545 nm, showing that the hemolysis rate of the PEG hydrogel groups was less than 1%. The hydrogel can interact with blood without negatively affecting blood cells or triggering undesirable responses, demonstrating its excellent blood compatibility.

Simultaneously, we conducted in vitro determination of the hydrogel’s blood clotting time using whole blood from rats. As shown in Figure 4e,f, the control group served as the untreated whole blood of rats, and we observed the blood clotting time to be approximately 313.4 ± 25.62 s by inclining the culture dish. In the experimental group, where PEG3 hydrogel covered the bottom of the culture dish, the time for blood clot formation was slightly shortened to 192.8 ± 14.55 s. Previous studies have reported zinc ions as crucial cofactors in thrombosis, capable of altering the structure of fibrin and reducing fibrinolysis. It is noteworthy that with the addition of 0.25% ZnO to the PEG3 hydrogel, the coagulation time was significantly reduced, forming a solid blood clot in approximately 1 min (60.2 ± 6.34 s). Therefore, the incorporation of ZnO into the PEG hydrogel significantly enhanced its procoagulant ability, providing a robust foundation for our subsequent in vivo hemostasis experiments [38,39].

### 2.6. In Vivo Hemostatic Performance

**Figure 5:**
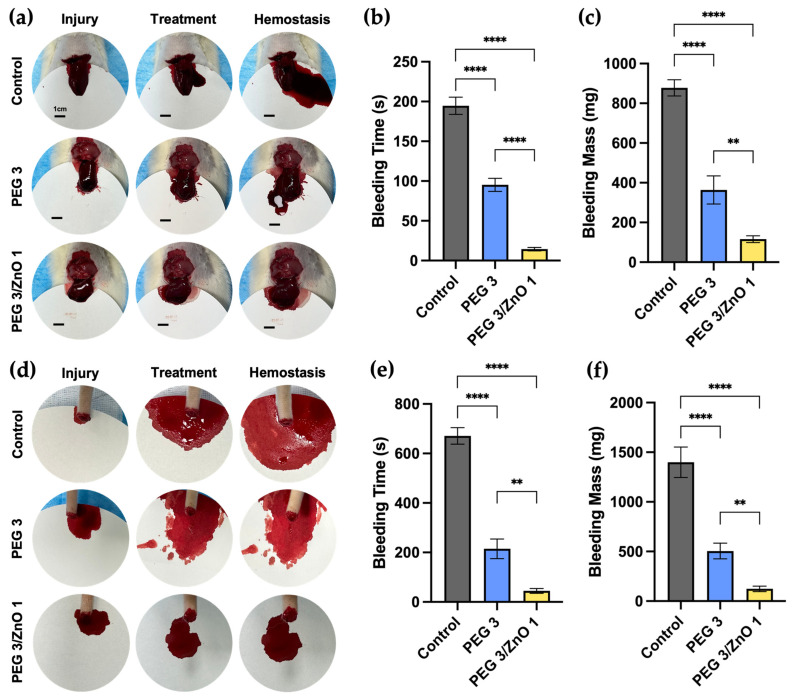
(**a**) Photograph of rat liver bleeding and hemostasis process (Scale bar = 1 cm). (**b**) Bleeding time in the liver hemorrhage (*n* = 3). (**c**) Bleeding mass in the liver hemorrhage (*n* = 3). (**d**) Photograph of rat tail bleeding and hemostasis process. (**e**) Bleeding time in the tail hemorrhage (*n* = 3). (**f**) Bleeding mass in the tail hemorrhage (*n* = 3). (Data are presented as mean ± SD, ** *p* < 0.01, **** *p* < 0.0001).

Based on the superior gelation speed, mechanical strength, biocompatibility, and in vitro pro-coagulant performance of PEG and PEG/ZnO hydrogels, we further investigated the in vivo adhesion and hemostatic capabilities of the hydrogels. We used rat liver bleeding and tail amputation bleeding models to simulate visceral and peripheral vascular bleeding, respectively. In the rat liver injury model (depicted in Figure 5a), a 1cm incision was made on the rat’s liver lobe, resulting in substantial bleeding. Subsequently, 400 μL of PEG3 hydrogel and PEG3/ZnO1 hydrogel were separately injected into the bleeding sites of the experimental group. As shown in Figure 5b,c, after injecting PEG3 hydrogel, a small amount of blood continued to flow out from the liver surface and mixed with the gel, turning the transparent hydrogel mixture red and gradually coagulating at the bleeding site. Compared to the control group, the bleeding time of PEG3 hydrogel decreased from 194.67 ± 8.81 s to 95.23 ± 6.72 s, and the bleeding mass decreased from 877.78 ± 33.24 mg to 363.88 ± 57.65 mg. In contrast, after injecting PEG3/ZnO1 hydrogel, the hydrogel rapidly diffused and solidified at the bleeding site, immediately stopping the bleeding. The coagulated hydrogel remained milky white, indicating that the hydrogel had already played a role in promoting coagulation before blood was mixed into the liquid hydrogel. Quantitative data also supported this view, as the bleeding time for PEG3/ZnO1 shortened to 14.43 ± 1.82 s, and the bleeding volume reduced to 115.84 ± 13.91 mg compared to the PEG3 group.

Similar results were obtained in the hemostasis experiment using the rat tail amputation bleeding model to simulate peripheral tissue bleeding (Figure 5d). As shown in Figure 5e,f, the untreated control group of rats had an average bleeding time of 671 ± 27.36 s and an average bleeding volume of 1399 ± 125.32 mg. After treatment with PEG3 and PEG3/ZnO1 hydrogels, the bleeding time for the experimental group of rats shortened to 214.67 ± 32.36 s and 44.67 ± 7.85 s, respectively. At the same time, the bleeding mass decreased to 504.33 ± 64.51 mg and 123.33 ± 21.79 mg, respectively. Both PEG and PEG/ZnO hydrogels can solidify on the wound surface through their own covalent reactions. Simultaneously, activated esters can capture amino groups in the tissue, further enhancing the gel’s adhesive properties. The combined action of these two steps played a role in physically sealing the wound surface. Furthermore, by releasing zinc ions, PEG/ZnO can activate crucial clotting enzymes, promoting platelet aggregation. This, in addition to the physical sealing provided by the PEG hydrogel, further facilitated blood clotting. These results indicated that PEG-based hydrogels exhibited excellent hemostatic performance and highlighted the effective procoagulant effect of adding ZnO, representing a promising approach for acute bleeding treatment.

## 3. Conclusions

In summary, this study represented a notable advancement in hemostatic material development, particularly focusing on PEG and PEG/ZnO hydrogels for efficient bleeding control in trauma and surgery. Through chemical analyses such as FT-IR and electron microscopy, the combination of PEG with ZnO has been shown to enhance the hydrogel’s mechanical strength, adhesion, and coagulation, resulting in improved structural integrity and accelerated gelation for practical clinical use. In vitro tests confirmed the biocompatibility and hemocompatibility of PEG/ZnO hydrogels, showcasing significant progress in compression and adhesion crucial for internal tissue stability. In vivo experiments with rat liver and tail bleeding models demonstrated superior hemostatic capabilities, significantly reducing bleeding time and volume compared to traditional PEG hydrogels. This promising approach offered a substantial improvement over conventional hemostatic methods, presenting enhanced mechanical properties and rapid gelation, paving the way for potential widespread clinical adoption and the prospect of saving lives in trauma and surgical scenarios, particularly in cases of severe bleeding and visceral injuries.

## 4. Materials and Methods

### 4.1. Materials

4-arm PEG-NH_2_ (Mw ≈ 10 K) and 4-arm PEG-SS (Mw ≈ 10 K) were purchased from SINOPEG. ZnO was purchased from Anhui Senrise Technology Co., Ltd. (Fuyang, China). All experimental materials are within their shelf life.

### 4.2. Characterization

Fourier transform infrared spectroscopy (FT-IR) was recorded on a Thermo Scientific Nicolet iS20 (Thermofisher, Waltham, MA, USA) in the range 400–4000 cm^−1^. X-ray photoelectron spectroscopy (XPS) was performed using VG ESCALAB 220 XL spectrometer (Thermofisher, Waltham, MA, USA) and XPS Peak-Fit 4.1 software to study the characteristic elements and their existing forms of the hydrogel and its components. Scanning electron microscopy (SEM) images were obtained at an acceleration voltage of 0.02–30 kV on a ZEISS Gemini 300 (ZEISS, Jena, Germany). The samples were directly sputter-coated with a thin layer and adhered to conductive adhesive. We used the Quorum SC7620 Sputter Coater to gold coat for 45 s, with the sputtering set at 10 mA. Subsequently, the SEM was employed for imaging the sample morphology with an accelerating voltage of 3 kV. The rheological behavior tests of the hydrogels were performed at room temperature. The prepared precursor solutions were placed in the Anton Paar MCR 302 rheometer (Anton Paar, Graz, Austria). During the time sweep, the hydrogels were tested under an angular frequency of 6.28 rad/s and a strain of 0.5%. In the strain sweep, the hydrogels were tested under an angular frequency of 10 rad/s.

### 4.3. Preparation of the Hydrogels

The solutions of PEG-NH_2_ and PEG-SS were separately prepared by dissolving predetermined proportions of each in PBS (pH 7.4) [12]. These solutions were then combined in equal volumes, followed by thorough mixing and vibration to form the PEG hydrogel. For the PEG/ZnO hydrogel, a specific amount of ZnO was dissolved in the PEG-SS solution to create the PEG-SS/ZnO solution. This was subsequently mixed in equal parts with the PEG-NH_2_ solution, undergoing a similar process of mixing and vibration, resulting in the formation of the PEG/ZnO hydrogel.

### 4.4. Gelation Time and Injectability

The determination of gelation times for both PEG and PEG/ZnO hydrogels was conducted using the tube inversion method. This involved mixing 200 μL of each PEG solution, after which the test tube was tilted to assess the fluid’s mobility [40]. The point at which the hydrogel ceased flowing and remained adhered to the tube’s bottom upon inversion marked the gelation time. Each hydrogel formulation underwent this test five times for consistency. Subsequently, 10 s after mixing the PEG-NH_2_ and PEG-SS (ZnO) solutions, the resulting liquid hydrogel precursor was extracted by the 21G needle syringe and then pushed out of the syringe to assess injectability. The ability of the hydrogel to be continuously injected before reaching the gelation state was considered good injectability [30].

### 4.5. Swelling Ratio and Degradation of the Hydrogels

Upon achieving the gelation state, each group of hydrogels was subjected to a freeze-drying process for 24 h and subsequently immersed in PBS. At predetermined time intervals, the hydrogels were extracted from the PBS solution, and surface moisture was meticulously blotted off with filter paper to ensure consistent weight measurements. The swelling ratio (SR) was calculated using the following formula:

$$SR\left(\%\right)=\frac{W_{t}-W_{d}}{W_{d}}\times 100\%$$

where W_t_ represents the weight of the hydrogels at various time points post-removal, and W_d_ denotes the weight of the hydrogels following freeze-drying. After a complete swelling period of 24 h, the hydrogels were extracted and weighed. This initial weight was recorded as W_0_. Subsequently, the hydrogels were immersed back into PBS, and at regular intervals, they were removed to be weighed again, with these subsequent weights marked as W_t_. The mass percentage was then calculated using the following formula:

$$Mass\left(\%\right)=\left(\frac{W_{t}}{W_{0}}\right)\times 100\%$$

where W_t_ represents the weight of the hydrogels at each extraction point, and W_0_ is the initial weight post-swelling.

### 4.6. Compression Strength Test

Ten minutes post-gelation, the cylindrical hydrogel samples were carefully removed. Their compression strength was then accurately assessed using a universal testing machine (Japan), operating at a steady compression speed of 2 mm/min until a compression level of 90% was achieved. This procedure was meticulously repeated three times for each hydrogel sample to ensure reliability and consistency in the measurements.

### 4.7. Adhesion Strength Test

Fresh porcine skin was meticulously cut into strips, each measuring 30 mm by 15 mm. A precise amount of 400 μL of hydrogel was then uniformly injected over a specific area of 15 mm by 10 mm on the edge of one strip of porcine skin. Subsequently, a second strip of porcine skin of identical size was carefully placed over the gel-coated area, ensuring proper alignment, and left undisturbed for 2 h to ensure adequate bonding. To measure the adhesion strength of the hydrogel, a universal testing machine (Japan) was employed, applying a tensile force at a consistent rate of 10 mm/min until the bonded skin samples separated, indicating sample fracture [41]. This process was rigorously repeated thrice for each hydrogel variant to guarantee accuracy and repeatability in the results.

### 4.8. Cytocompatibility and Fluorescence Staining

To evaluate the impact of hydrogels on cellular proliferation, both CCK-8 assays and fluorescence staining were conducted. NIH-3T3 cells were initially grown in Dulbecco’s Modified Eagle Medium (DMEM), supplemented with 10% fetal bovine serum and 1% penicillin/streptomycin, sourced from Biological Industries, Israel. After a 24 h culture period, the cells were evenly distributed into 96-well plates at a concentration of 2 × 10⁴ cells per well. Following a further 24 h incubation in a humidified 5% CO_2_ environment, the medium was replaced with a hydrogel-conditioned medium, which was prepared by a ratio of 1 g hydrogel to 10 mL complete culture medium and co-culturing for 24 h. Cell proliferation was then measured at intervals of 1, 2, and 3 days at 37 °C using the CCK-8 assay. This process was rigorously repeated five times for each hydrogel to ensure reliable results. The absorbance of the sample solutions was recorded at 450 nm, and cell viability was calculated as follows:

$$Cell\;Viability\left(\%\right)=\frac{{OD}_{sample}-{OD}_{blank}}{{OD}_{control}-{OD}_{blank}}\times 100\%$$

where OD_sample_ represents the absorbance of the hydrogel group, OD_control_ is the absorbance of the control group in complete medium, and OD_blank_ refers to the blank well’s absorbance. Concurrently, after 1 day of incubation, cells were stained with DAPI and phalloidin (both from Solarbio, Beijing, China) to visualize the nuclei and actin proteins, respectively.

### 4.9. Hemocompatibility

For hemolysis assessment, 0.4 g of both PEG and PEG/ZnO hydrogels was immersed in 4 mL of PBS for 12 h to create hydrogel extracts. Fresh rat whole blood was diluted with 5 mL PBS to form a diluted solution. Subsequently, 500 μL of this diluted blood was added to 3 mL of each hydrogel extract, as well as to PBS and 0.1% Triton X-100, serving as negative and positive controls, respectively. These mixtures were then incubated at 37 °C for 12 h. Post-incubation, the supernatants were separated by centrifuging at 4000 rpm for 3 min. The absorbance of these supernatants was measured at 540 nm to determine hemolysis levels [42]. The hemolysis ratio was calculated with the following formula:

$$Hemolysis\left(\%\right)=\frac{{OD}_{sample}-{OD}_{negative}}{{OD}_{positive}-{OD}_{negative}}\times 100\%$$

where OD_sample_ is the absorbance of the hydrogel extract, OD_positive_ is the absorbance of the purified water group, and OD_negative_ is that of the PBS group. Each hydrogel was tested 5 times for consistency.

### 4.10. In Vitro Clotting Time

A total of 100 μL of the prepared hydrogel precursor solution was injected to cover the bottom of the 96-well plate. Then, 100 μL of citrated rat blood mixed with 5 μL of calcium chloride (CaCl_2_) was added into both the blank and hydrogel-covered wells. The plate was periodically tilted, and the wells were washed every minute with PBS to remove uncoagulated blood. The clotting time was recorded, defined as the duration when there was no significant flow of blood upon tilting the culture plate and the blood clot could not be washed away by PBS [21]. Each hydrogel was tested 5 times for consistency.

### 4.11. In Vivo Hemostatic Performance

The assessment of hemostatic efficacy was conducted on rat liver and tail hemorrhage models using eighteen 8-week-old female Sprague-Dawley rats, each weighing approximately 200 ± 15 g [43]. These subjects were randomly categorized into three distinct groups: control, PEG, and PEG/ZnO. Administered intraperitoneally with 2% *w*/*v* pentobarbital sodium, the rats were anesthetized and securely positioned on the surgical table. Following the preparation of the operative area, which involved shaving the fur, a 1.5 cm horizontal incision was precisely made beneath the xiphoid process. This incision penetrated the epidermal layer, subcutaneous tissue, and fascia. To arrest the bleeding, the area was adequately compressed with gauze, revealing the left hepatic lobe. Excess intra-abdominal fluid was then meticulously aspirated using gauze. A pre-measured piece of filter paper was strategically placed under the liver for accurate blood collection. A subsequent transverse cut, measuring 10 mm in length and 5 mm in depth, was executed on the liver. The cranial aspect of the rat was slightly elevated, and 400 μL of hydrogel was precisely administered to the hemorrhagic site for the treatment groups, whereas the control group received no intervention. Once the cessation of active bleeding was confirmed, the filter paper was weighed to accurately record both the volume and duration of the bleeding episode. In the tail hemorrhage model, the distal third of the rat’s tail was amputated. Here, 200 μL of hydrogel was applied to the bleeding site, and blood loss was collected on a pre-weighed piece of filter paper. After the cessation of active bleeding, the paper was weighed, facilitating the quantification of both the total bleeding volume and time. To ensure the consistency and reproducibility of results, each hydrogel formulation was subjected to three independent tests.

### 4.12. Statistical Analysis

All gathered experimental data were meticulously computed and presented as mean ± standard deviation (SD). Statistical analysis of the data was performed using one-way ANOVA, followed by Tukey’s post hoc test to determine the significance of differences among groups. In this statistical framework, a *p*-value of less than 0.05 (* *p* < 0.05) was considered to indicate a statistically significant difference.

## Data Availability

The data presented in this study are available in this article.
